# Rationale of Cruciate Retaining Design in Rheumatoid Arthritis: A Review of Clinical Analysis and its Role in Rheumatoid Arthritis

**DOI:** 10.2174/1874325001711011023

**Published:** 2017-08-29

**Authors:** Munis Ashraf, Om Prakash Sharma, Sruthi Priyavadhana, Senthil Nathan Sambandam, Varatharaj Mounasamy

**Affiliations:** Department of Orthopedic Surgery, KG Hospital and Post Graduate Medical Institute. Coimbatore, Tamil Nadu, India

**Keywords:** Rheumatoid arthritis, Cruciate retaining, Posterior Stabilised, Total Knee Arthroplasty, Survivorship, Functional Outcome

## Abstract

**Background:**

Over the years, proponents of total knee designs (cruciate retaining and posterior stabilised) have conducted several long-term studies to claim the potential of these designs in several subsets of patients. Total knee arthroplasty (TKA) in patients with rheumatoid arthritis has also been one such domain where numerous studies were conducted in the past. A general perception among majority of arthroplasty surgeons is that, posterior stabilised (PS) is the implanted design of choice among patients with Rheumatoid arthritis (RA). However, with the available literature there is a significant disparity related to the selection of implants in patients with rheumatoid RA. In this review of literature, an attempt is made to identify the clinical performance and role of one such implant design, the cruciate retaining (CR) prosthesis in rheumatoid arthritis.

**Method:**

The review was conducted after a series of advanced search in the following medical databases; Pub med, Biomed central, Cochrane and Google scholar for articles related to long term follow up studies of cruciate retaining total knee arthroplasty in rheumatoid arthritis using the keywords cruciate retaining prosthesis, total knee arthroplasty, rheumatoid arthritis.

**Results:**

The available data demonstrate that the CR design is attributed with an excellent long term survivorship and functional outcome even in follow up studies up to twenty-five years.

**Conclusion:**

The advantages of using a CR design are long term survivorship, controlled femoral roll back and preservation of bone stock. Thus, the data gathered in this review lead to a consideration that the CR design is an implant design on par with PS design in patients with RA.

## 1 INTRODUCTION

It is estimated that 25% of Rheumatoid arthritis (RA) patients will undergo total joint arthroplasty (TJA) within 21.8years of disease onset [1]. Total knee arthroplasty [TKA] is a largely successful procedure which alleviates pain and improves quality of life in patients with degenerated and deformed joints. Rheumatoid arthritis is a systemic disease that also affects the large joints, the knee joint is most commonly affected and there occurs severe symmetrical joint space reduction in a relatively younger population. Unlike total knee arthroplasty in primary osteoarthritis, patients with RA have an ongoing systemic disease which is progressive and pre-disposes to soft tissue damage and poor bone quality. This can thereby lead to altered biomechanics. The presence of these articular and extra articular pathologies makes the selection of implants imperative for a long-term survivorship in these patients. In the absence of a valid consensus regarding implant selection, a skewed approach of using PS implants in every patient with RA has been adopted by majority of the surgeons without proper patient selection. This review analyses the clinical performance, long term survivorship of cruciate retaining (CR) design, which is considered as an “outlier” in such patients.

## 2 METHODOLOGY

The review was conducted after a series of advanced search in the following medical databases; Pub med, Biomed central, Cochrane and Google scholar for articles related to long term follow up studies of cruciate retaining total knee arthroplasty in rheumatoid arthritis using the keywords cruciate retaining prosthesis, total knee arthroplasty, and rheumatoid arthritis.

## 3 REVIEW OF CLINICAL ANALYSIS

### 3.1 Long Term Survivorship

Patients with Rheumatoid arthritis usually present for TKA at a younger age when compared with primary osteoarthritis, approximately ten years younger [2]. Hence the survival of the implant plays a major role in these patients. The data accumulated from heterogenous centres have determined the survivorship analysis using Kaplan Meier, Cutler Ederer and Armitage actuarial methods. In this review, survivorship of the implant was based on the need for re operation for any reason.

 From the studies listed in Table **1**, it is evident that the ten-year survival of CR in RA is comparable to that of the CR in OA knees which is 90% as evidenced by Abdel et el in 5389 cruciate retaining TKA [3]. Furthermore, it was summarised that the most durable result is following a cruciate retaining prosthesis in an elderly woman with inflammatory arthritis by Rand *et al.* in a large cohort of 11,606 knee arthroplasties [4].

### 3.2 Functional Outcome

A long-term assessment functional outcome assessment in patients with RA is essentially required considering the relatively younger population present for TKA. Among the studies included in this article, the functional outcome assessment had been made using the knee society functional outcome score and hospital for special surgery score. One of the earliest studies by Gill and Joshi demonstrated excellent functional outcome scores only in 15% of knees at final follow up [5]. With time, the design of cruciate retaining had evolved and the functional outcomes have improved ever since.

 This change is noted in several studies across the globe, a 25 year follow up study from Mayo clinic demonstrated a good functional outcome score (mean score 79) at the end of 25 years [6, 7]; a minimum 15 year follow up study from Korea noted an excellent post-operative functional score of 80 at final follow up [8]; a 12 year follow up study from Japan shows a good mean functional outcome score of 70 at final follow up [9]. These findings further reiterate the favourable long term functional status of cruciate retaining prosthesis.

### 3.3 Proprioception

A posterior cruciate ligament (PCL) with intact neural structures aids in proprioception, whereas a PCL deficient knee leads to instability and early degenerative changes in the knee [10]. The structural integrity of posterior cruciate ligaments was assessed by a histopathological study which demonstrated the intactness of PCL in RA patients with grade 3 or grade 4 of the disease [11]. The findings suggest that a careful intra-op assessment of PCL integrity is required and sacrificing the PCL without proper patient selection is not ideal.

### 3.4 Range of Motion

Cruciate retaining design is a less constrained prosthesis which allows better controlled femoral roll back than posterior stabilised design. Among the available literature Table **1**, the maximum average range of motion of 115^o^ was noted in a 22 year follow up study by Lee *et al.* and correlates with the average knee ROM after TKA in OA patients [8]. The longest follow up study till date from Mayo clinic demonstrates an average knee ROM of 100^o^ [6, 7]. Considering the reduced ambulatory status in RA, an average ROM of 100^0^ would improve the mobility and quality of life substantially.

## 4 REVIEW OF COMPLICATIONS SPECIFIC TO CRUCIATE RETAINING IN RHEUMATOID ARTHRITIS

### 4.1 Failure Due to Instability

It is a popular belief to use a more constrained design such as a PS implant in patients with RA, this was largely based on a 6-year follow up study from the Hospital for special surgery, New York which reported a 50% incidence of posterior instability in cruciate retaining design leading to revision [12]. However, apart from this isolated study which indicates such a high rate of instability using CR design; there are numerous long term outcome studies from multiple centres which disapproves of this fallacy.

 The role of CR design with regards to posterior instability in RA patients is evidenced in the following studies, Miller *et al.* [7] in a 25 year follow up study reported that only two patients required re-operation due to instability; Gill and Joshi [5] evidenced that one patient (1\66) required re-operation for posterior instability; Belleman *et al.* [13] had documented that there is no statistically significant difference between CR and PS with regards to posterior instability in his long term study. Additionally, biomechanical studies have demonstrated the role of PCL in varying degrees of flexion and its importance as a primary restraint against posterior translation in varying angles of flexion [14, 15].

### 4.2 Aseptic Loosening

Component loosening after total knee arthroplasty is a cause for revision and occurs usually due to an increased polyethylene wear debris generated, which triggers an immune response leading to resorption of the bone-implant interface. In rheumatoid arthritis, there is a theoretical risk of accelerated resorption of bone-implant interface due to a reactivation of synovitis [16]. An isolated study has reported the incidence of revision due to aseptic loosening to be in 3 (4.5%) out of 66 knees in which cruciate retaining prosthesis was used [5]. Apart from this study, long term studies with a follow up period of up to 25 years have clearly evidenced that the survival rate of implant at the end of ten years, fifteen years and twenty five years was 100% with component loosing due to aseptic causes as the end point [6-9, 17].

### 4.3 Periprosthetic Fracture

Rheumatoid arthritis is a progressive disease leading to symmetrical joint space reduction and secondary arthritis. In addition, chronic steroid use leads to osteopenia which subsequently leads to a poor bone quality. Archibeck had reported one case of peri prosthetic fracture using a CR design out of 72 knees [6]. The use of PS design demands an obligatory box cut which further weakens the intercondylar notch in a patient with a small sized femur and leads to a theoretical risk of peri prosthetic fracture [18]. Hence it is imperative that bone resection is minimal during knee arthroplasty.

## CONCLUSION

The current trend in preferential use of PS design in cases with RA among the surgeons was largely influenced by isolated studies which demonstrated failure due to instability in CR design and a deficient posterior cruciate ligament in these patients. However, homogenous data from heterogenous centres which has been accumulated in this review is rather on the contrary to the general perception of using PS in every case of rheumatoid arthritis. The available data demonstrates that the CR design has an excellent long term survivorship and functional outcome even in follow up studies up to twenty-five years. The advantages of using a CR design are long term survivorship, controlled femoral roll back and preservation of bone stock. Thus, the data gathered in this review leads to a consideration that the CR design is an implant design on par with PS design in patients with RA.

## Figures and Tables

**Table 1:** List of studies related to cruciate retaining in rheumatoid arthritis.

	Study	Published year	Prosthesis	follow up Period	Range of Motion (avg)	Survivorship*
1.	Gurdev & Gill	2000	CR	0.2 to 19.8	106^o^	85.2%(10 years)
2.	Bellmans *et al.*	1997	CR	3 to 13 years	90^o^	97% (5 years)
3.	Schai *et al.*	1998	CR	10 to 13 years	113^o^	90% (10 years)
4.	Archibeck *et al.*	2001	CR	8 to 14 years	104^o^	81% (10 years)
5.	Miller *et al.*	2011	CR	20 to 25 years	100^o^	69% (20 years)
6.	Lee *et al.*	2012	CR	9 to 22 years	115^o^	98.7% (10 years) 83.6% (17 years)
7.	Yamanaka **et al.*.*	2012	CR	5 to 12 years	110^o^	96.9% (12 years)

‘*’ END POINT BEING REVISION FOR ANY CAUSECR- Cruciate retaining.

## LIST OF ABBREVIATIONS

RA: = Rheumatoid arthritis
TJA: = Total Joint arthroplasty
TKA: = Total knee arthroplasty
CR: = Cruciate retaining
PS: = Posterior stabilised
PCL: = Posterior cruciate ligament
